# Dose-Dependent Protective Effect of Bisperoxovanadium against Acute Cerebral Ischemia in a Rat Model of Ischemia/Reperfusion Injury

**DOI:** 10.3390/ijms140612013

**Published:** 2013-06-05

**Authors:** Jian-Yi Guo, Jun Ding, Fang Yuan, Hao Chen, Shi-Wen Chen, Heng-Li Tian

**Affiliations:** Department of Neurosurgery, Shanghai 6th People′s Hospital, Shanghai Jiaotong University, Shanghai 200233, China; E-Mails: jianyiguo@gmail.com (J.-Y.G.); dingjun198408@126.com (J.D.); yf021025@sjtu.edu.cn (F.Y.); chenhao_316@yahoo.com.cn (H.C.); chenshiwen@126.com (S.-W.C.)

**Keywords:** bisperoxovanadium, phosphatase and tensin homologue deleted on chromosome 10, cerebral ischemia, neuroprotection, Akt

## Abstract

PTEN (phosphatase and tensin homologue deleted on chromosome 10) is a dual-specificity lipid and protein phosphatase. The loss of PTEN was originally discovered in numerous human cancers. PTEN inhibition by bisperoxovanadium (bpV) reduces neurological damage after ischemic brain injury. The purpose of this study was to identify the optimal neuroprotective dose of bpV when administrated after focal ischemia/reperfusion (I/R) injury in rats. Focal I/R injury was induced using the middle cerebral artery occlusion method. bpV at doses of 0.25, 0.50 and 1.0 mg/kg were injected intraperitoneally just after reperfusion, with saline serving as a vehicle control. A maximal reduction in brain injury was observed with 1.0 mg/kg bpV. This dose of bpV also significantly blocked apoptosis in the penumbral cortex of rats. This beneficial effect was associated with the increasing levels of Akt phosphorylation in the penumbral cortex. These results demonstrate that the pharmacological inhibition of PTEN protects against I/R injury in a dose-dependent manner and the protective effect might be induced through upregulation of the phosphoinositide-3 kinase/Akt pro-survival pathway, suggesting a new therapeutic strategy to combat ischemic brain injury.

## 1. Introduction

Stroke remains a major cause of long-term disability and death in adults [1], yet few therapeutic approaches are available to treat patients. Among the available options, such as thrombolytic tissue plasminogen activator (tPA), a narrow therapeutic window limits their usefulness in the clinic [2]. Therefore, it is essential that new therapies be developed to improve stroke outcome.

PTEN (phosphatase and tensin homologue deleted on chromosome 10) is a tumor-suppressor gene localized to chromosome 10q23. Loss of PTEN is common in a large number of human cancers, including glioblastomas, melanomas, and endometrial and prostate carcinomas [3]. In the central nervous system, PTEN is involved in multiple aspects of normal function, including neuronal migration [4] and neuronal size and structure control [5], as well as in pathological processes of neuronal injury, including those associated with brain ischemia and neurological disorders such as human Lhermitte-Duclos disease and drug addiction [6–8]. It has been suggested that downregulating PTEN activity may have potential therapeutic effects reducing brain injury in various experimental models [9–11]. Bisperoxovanadium (bpV) is a well-established inhibitor of protein tyrosine phosphatases that effectively inhibits PTEN at low concentrations [12]. However, the dosage effect of the drug has not yet been studied. The purpose of this study was to identify the optimal neuroprotective dose of bpV in cerebral ischemic injury and to investigate whether this possible neuroprotection is associated with reduced apoptosis and signaling pathway activation in the penumbral areas of ischemic brain.

## 2. Results and Discussion

### 2.1. Effect of Bisperoxovanadium (bpV) on Infarct Volume

**Figure 1:**
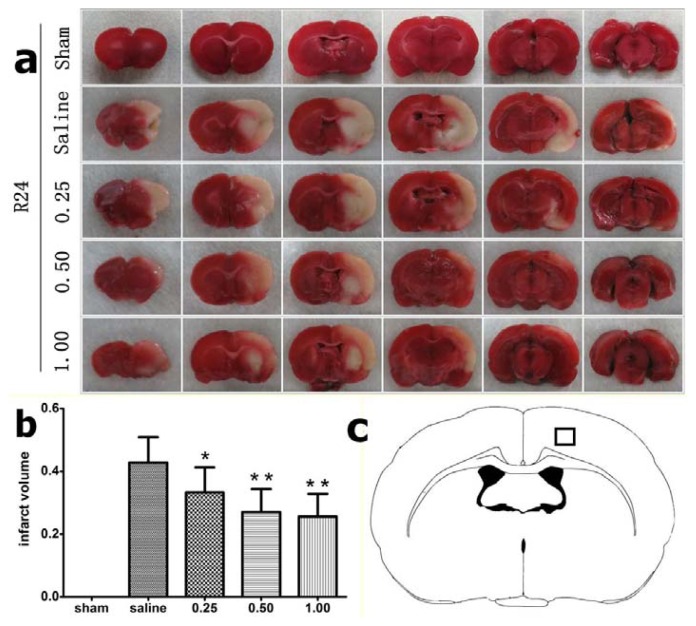
(**a**) Coronal sections of rat brain, 2 mm thick, stained with 2,3,5-triphenyltetrazolium chloride (TTC). Non-ischemic areas appear red, and ischemic areas appear white. Note the decrease in ischemic area of rats treated with bisperoxovanadium (bpV); (**b**) Quantitative analysis of cerebral infarct volume in each group (*n* = 9). Data are expressed as the means ± SD. * *p* < 0.05, ** *p* < 0.01 compared with the saline group; (**c**) The outlined region was designated as the penumbral cortex.

To evaluate the protective effect of bpV on cerebral I/R injury, we treated rats with 0.25, 0.5, and 1 mg/kg bpV. None of the animals was dead within 24 h after reperfusion. At 24 h after reperfusion, the rats were sacrificed and TTC staining for infarct volume assessment was performed. The relative infarct volume in saline-treated animals was 42.81% ± 8.07%. Administration of bpV at doses of 0.25 mg/kg (infarct volume, 32.17% ± 7.99%, *p =* 0.013), 0.50 mg/kg (27.04% ± 7.27%, *p =* 0.000), and 1.00 mg/kg (25.56% ± 7.25%, *p =* 0.000) significantly decreased infarct volume by 24.85%, 36.84%, and 40.29%, respectively (Figure 1). The maximum effect was observed with 1.0 mg/kg bpV, suggesting dose-dependent protection by bpV with respect to infarct volume in the rat I/R model. In subsequent experiments, we chose a dose of 1.0 mg/kg body weight to study the protection by bpV.

### 2.2. Effects of bpV on Neurological Deficits

**Figure 2:**
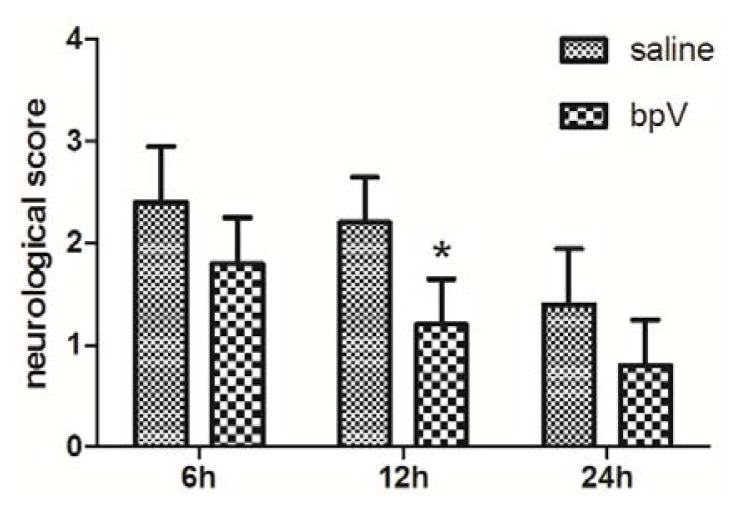
Neurological deficits scores of both bisperoxovanadium (bpV)- and saline-treated animals 6, 12 and 24 h after reperfusion. Neurological deficits were significantly ameliorated in rats treated with bpV compared with saline-treated controls at 12 h after reperfusion, but not at 6 or 24 h after reperfusion. *n* = 5, * *p* = 0.015.

Neurological deficits were assessed 6, 12, and 24 h after reperfusion (evaluation system presented in Section 3.4). The groups treated with bpV exhibited remarkably reduced neurological deficit scores compared with the saline-treated group at 12 h after reperfusion (Figure 2, *p* < 0.05). However, at 6 and 24 h after reperfusion, no significant difference between the groups was found (Figure 2, *p* > 0.05).

### 2.3. bpV Decreased Neuron Apoptosis Induced by Cerebral Ischemic/Reperfusion Injury

**Figure 3:**
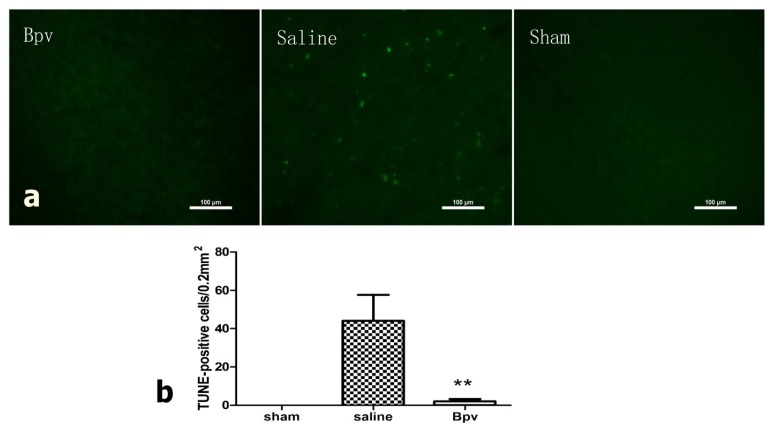
Bisperoxovanadium (bpV) administration blocks TUNEL-positive staining in the penumbral cortex 24 h after ischemia/reperfusion injury. (**a**) Photomicrographs of TUNEL-positive cells in the penumbral cortex. Scale bar = 100 μm; (**b**) Bar graphs of TUNEL-positive cell counts in each group. *n* = 5, ** *p* = 0.001.

Induction of apoptosis was quantified by assessing TUNEL-positive cells in penumbra 24 h after reperfusion, as shown in Figure 1c. TUNEL-positive cells were not observed in sham-operated animals (Figure 3). In saline-treated animals that underwent I/R injury, cells in the penumbral cortex were strongly positive for TUNEL staining. This effect was not observed in bpV-treated animals (*p* < 0.01).

### 2.4. Effect of bpV on Phosphorylation of Akt (Ser473)

**Figure 4:**
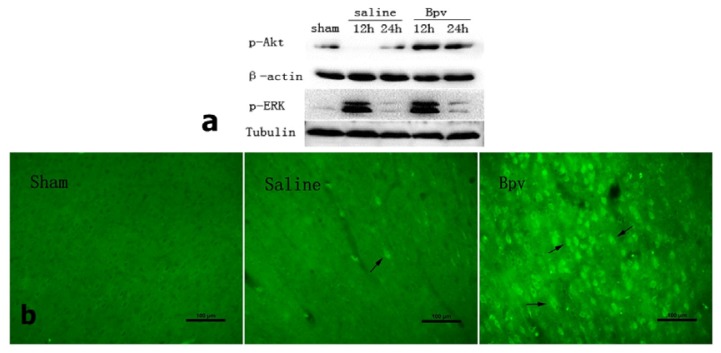
(**a**) Representative Western blots for p-Akt (Ser 473) and p-ERK1/2 with β-actin and tubulin serving as a loading control. *n* = 5; (**b**) Photomicrographs of p-Akt (Ser473) in the penumbral cortex in sham, saline-treated, and bpV-treated animals. The arrow indicates p-Akt (Ser473)-positive cells. Scale bar = 100 μm, *n* = 3.

To investigate whether PI3K/Akt is involved in the neuroprotective effect of bpV, we performed Western blot analysis to assess the phosphorylation of Akt (p-Akt, Ser 473) in penumbra area. bpV significantly increased p-Akt (Ser 473) compared with the saline group (Figure 4a). p-ERK served as a positive control. Consistent with previously reported findings [13], our results showed that levels of p-ERK1/2 increased early and then declined to near the levels seen in operated animals. We also examined immunoreactivity in the penumbral cortex 12 h after reperfusion, where p-Akt immunoreactivity was markedly increased (Figure 4b).

### 2.5. Discussion

In this study, our results indicate that administration of bpV at doses of 0.25, 0.50 and 1.0 mg/kg effectively reduced brain damage by 24.85%, 36.84%, and 40.29%, respectively. However, there was no significant difference between the 0.5 and 1.0 mg/kg groups (*p* > 0.05), indicating that the protective effect of bpV reached a plateau and increasing the drug dose would not have a greater protective effect.

The optimal dose, 1.0 mg/kg bpV, produced a neuroprotective effect that resulted in reduced cell apoptosis and significantly increased p-Akt activity in the penumbral cortex. bpV treatment also improved neurological scores at 12 h, but not at 24 h, after reperfusion. This result is congruent with previous studies demonstrating reduced infarct volumes and improved functional outcome [14,15].

The majority of delayed neuronal degeneration is due to apoptosis. Results showed fewer TUNEL-positive cells in bpV-treated than in saline-treated rats. Activation of the PI3K/Akt pathway is critical for neuroprotection from ischemia-induced apoptosis. To determine whether Akt activation contributes to PTEN inhibition against apoptosis induced by I/R injury, we investigated the phosphorylation of Akt by Western blot. Treatment with bpV significantly increased p-Akt (Ser473), compared with the saline-treated group. Further immunohistochemistry staining showed that p-Akt (Ser473) in the penumbral cortex was markedly increased.

Most cells undergoing apoptosis were located in the penumbral cortex, and it is likely that apoptotic neurons are responsible for successive injury. By salvaging these apoptotic neurons, the pathological outcome could be improved. Activated Akt blocked apoptosis by phosphorylating and thus suppressing most of its substrates, such as Bcl-associated death protein, forkhead transcription factor, and glycogen synthase kinase 3 [16–18]. Our study showed that bpV at a dose of 1.0 mg/kg decreased neuronal apoptosis in the penumbral cortex (Figure 3) and increased p-Akt (Ser-473) in treated rats compared with rats in the sham group.

PTEN is a dual-specificity lipid and protein phosphatase extensively studied in cancers. Recently, there has been increasing interest in the role of PTEN in cellular function, particularly in neurons. Studies suggest that PTEN regulates cell viability and susceptibility to apoptosis by negatively regulating the PI3K/Akt pathway [19]. An *in vitro* study showed that PTEN deletion promoted axon regeneration and functional repair after adult spinal cord injury [20]. Another study showed that reduced PTEN levels in mouse hippocampal neurons were more resistant to seizure-induced cell death compared with wild-type littermates [21]. Further *in vivo* studies demonstrated that phosphorylated PTEN levels were altered in ischemic penumbral regions, thereby regulating Akt activity [8]. PTEN inhibition generates similar protective effects in other organs. For example, PTEN gene knockdown induced cardioprotection against I/R injury in isolated mouse hearts [22]. In addition, inhibition of PTEN promoted the generation of induced pluripotent stem cells derived from mouse somatic cells [23]. In retinal ganglion cells, simultaneous deletion of both PTEN and SOCS3 enables sustained axon regeneration [24]. Thus, downregulating PTEN activity may represent a novel pharmacological approach for treatment of ischemia.

## 3. Experimental Section

### 3.1. Ischemic Stroke Model

All animal experiments were approved by the Ethics Committee of Laboratory Animal Welfare at Shanghai Jiaotong University, Shanghai, China. Middle cerebral artery occlusion (MCAO) surgery was performed using the monofilament suture method [25] in male Sprague-Dawley rats weighing 250–280 g, after anesthetized with 10% chloral hydrate (400 mg/kg, intraperitoneally). A 4-0 poly-l-sine-coated monofilament nylon suture with a round tip (Beijing Sunbio Biotech Co. Ltd., Beijing, China) was introduced into the internal carotid artery through a nick given in the external carotid artery and advanced 18–20 mm from the common carotid artery bifurcation to block the origin of middle cerebral artery. Sixty minutes after occlusion, each rat was tested. Rats that did not show initial contralateral paralysis like failure to extend left forepaw or circling to the left were excluded from further study. Two hours after the MCAO procedure, rats were re-anesthetized, and the intraluminal sutures were gently withdrawn to restore blood supply to the MCA region. Throughout the procedure, body temperature was maintained at 37 ± 0.5 °C with a homothermal blanket. A sham group of animals received the same surgical procedure, but the filament was advanced for only 10 mm and immediately withdrawn. After recovery from anesthesia, animals were kept in individual cages with free access to food and water.

### 3.2. Bisperoxovanadium (bpV[HOpic]) Treatment

Rats were completely randomized into groups. A total rats of 39 were subjected to MCAO surgery and 3 rats were excluded due to the lack of contralateral paralysis. Immediately after reperfusion, 36 rats (*n* = 9 per group) were randomly treated with intraperitoneal doses of bpV[HOpic] (ENZO Life Sciences, Farmingdale, NY, USA) at 0.25, 0.5 or 1.0 mg/kg or with 0.9% saline vehicle. The drug was dissolved in vehicle immediately before injection to a total volume of 1 mL. All animals were sacrificed for TTC staining 24 h after reperfusion.

In subsequent experiments, 36 rats (41 rats were subjected to surgery and 5 rats excluded for the reason mentioned above) were treated with the dose of bpV that produced a maximal reduction in cerebral infarction or saline to study neurological deficits, neuron apoptosis, and activation of signaling pathways. Rats were completely randomized to different treatment and tests. The animals were sacrificed 12 or 24 h after reperfusion.

All of the evaluation including infarct size, neurological deficits, neuron apoptosis, western blot and immunohistochemistry were performed by an investigator who was blind to the experimental groups.

### 3.3. Infarct Volume Assessment

Brains were removed immediately after sacrifice by an overdose of chloral hydrate and rapidly transferred to −80 °C for 4 min. Each brain was sliced into 2-mm coronal sections. The slices were incubated with 2% 2,3,5-triphenyltetrazolium chloride (TTC) solution (Sigma, Shanghai, China) for 15 min at 37 °C and fixed in 10% buffered paraformaldehyde. The unstained areas, which appeared pale, were defined as infarct regions, and areas that appeared red were defined as normal. TTC-stained slices were photographed separately using a digital camera (Canon, Tokyo, Japan) and analyzed using Photoshop (CS3 Adobe, San Jose, CA, USA). The relative lesion volume was calculated as described previously [26]. The infarct volume of each slice was determined by multiplying the area times the slice thickness (2 mm). The volumes of each slice were then summed to determine the whole infarct volume of each brain. The contribution of edema to the infarct volume was achieved using the following equation as previously described [27]:

Corrected infarct volume=left hemisphere size-(right hemisphere size-measured infarct size)

### 3.4. Neurological Deficit Evaluation

Evaluation of neurological deficits was performed 6 h, 12 h and 24 h after reperfusion by using a five-point scoring system described previously [25]: no neurologic deficit = 0, failure to extend left forepaw fully = 1, circling to the left = 2, falling to the left = 3, and no spontaneous walking accompanied by a depressed level of consciousness = 4.

### 3.5. Terminal Deoxynucleotidyl Transferase dUTP Nick End Labeling (TUNEL)

To detect spatial distribution of stroke-caused DNA fragmentation, a TUNEL staining protocol was employed with minor modifications [28]. Five rats in each goup were sacrificed for TUNEL staining 24 h after reperfusion Under deep anesthesia, rats were perfused transcardially with phosphate-buffered saline (PBS) followed by 4% paraformaldehyde. The brains were removed, kept in 4% paraformaldehyde overnight, and then immersed in 30% sucrose for 3 days at 4 °C. After embedding in OCT (SAKURA, Tissue-Tek, Torrance, CA, USA), cryostat-cut 40-μm sections were subjected to the fluorescent TUNEL technique. Sections were first incubated in a permeabilization solution (0.1% Triton X-100 in 0.1% sodium citrate) for 30 min on ice and rinsed twice with PBS. The sections were then incubated with a mixture of TdT enzyme (Promega, Madison, WI, USA) and biotinylated 16-dUTP (Roche) diluted in the TdT buffer (Promega, Madison, WI, USA) at 37 °C for 60 min, followed by washing twice in PBS for 15 min. Sections were then incubated with avidin-488 for 60 min at room-temperature. TUNEL stained sections were examined under a fluorescence microscope (Nikon, 80i, Tokyo, Japan). Five areas of penumbra cortex (Figure 1c) in each slice were examined under 100× magnification and TUNEL-positive cell numbers were counted.

### 3.6. Western Blot Analysis

At 12 h and 24 h after reperfusion, 5 rats in each goup were sacrificed for western blot. Penumbra cortex samples for Western blot analysis were prepared as described previously [29]. The tissue samples were homogenized and centrifuged at 12,000× *g* for 15 min at 4 °C. Supernatant was collected and protein concentration was determined by the modified Lowry assay. Equal amounts of protein per lane were separated by 10% sodium dodecyl sulfate (SDS)-polyacrylamide gel electrophoresis (PAGE) and transferred to polyvinyldifluoridine membrane. The membranes were incubated overnight at 4 °C with primary antibodies against phosphorylated Akt (p-Akt-Ser473; 1:1000, Cell Signaling, Danvers, MA, USA), and β-actin (1:200) followed by horseradish peroxidase-conjugated anti-rabbit or anti-mouse secondary antibodies (1:1000, Amersham Biosciences, Pittsburgh, PA, USA). The protein bands were visualized using the Tanon image system (Tanon2500R, Shanghai, China) and scanned. The relative band densities were analyzed using ImageJ (1.44P, NIH).

### 3.7. Immunohistochemistry

Three rats in each group were sacrificed for immunohistochemistry 12 h after reperfusion. Tissue sections were fixed in 4% paraformaldehyde, as described above. Sections were incubated with primary antibodies against p-Akt-Ser473 (1:1000, Cell Signaling) at 4 °C overnight. Sections were washed with PBS and incubated with Alexa Fluor 488 goat anti-rabbit IgG for 2 h at room temperature and protected from light. Fluorescence was detected with a fluorescence microscope.

### 3.8. Statistical Analysis

The data are presented as means ± standard deviation (SD). One-way analysis of variance (ANOVA) with post hoc test (LSD test) were used to assess differences among multiple groups. Comparisons between two groups were assessed using Student′s *t*-test. Mann-Whitney *U* test was used to assess non-parametric statistics. Differences were considered significant when *p* < 0.05.

## 4. Conclusions

Our study demonstrated that the PTEN-inhibitor bpV provided dose-dependent neuroprotection in rats following I/R injury. The underlying mechanism might be reduced apoptosis in penumbral area through the PI3K/Akt pathway. Additional studies will be required to fully understand the biochemical mechanisms underlying PTEN-mediated pathways in cerebral ischemia disease.

## References

[1] Zaleska M.M., Mercado M.L., Chavez J., Feuerstein G.Z., Pangalos M.N., Wood A. (2009). The development of stroke therapeutics: Promising mechanisms and translational challenges. Neuropharmacology.

[2] Hacke W., Brott T., Caplan L., Meier D., Fieschi C., von Kummer R., Donnan G., Heiss W.D., Wahlgren N.G., Spranger M. (1999). Thrombolysis in acute ischemic stroke: Controlled trials and clinical experience. Neurology.

[3] Li J., Yen C., Liaw D., Podsypanina K., Bose S., Wang S.I., Puc J., Miliaresis C., Rodgers L., McCombie R. (1997). PTEN, a putative protein tyrosine phosphatase gene mutated in human brain, breast, and prostate cancer. Science.

[4] Leslie N.R., Yang X., Downes C.P., Weijer C.J. (2005). The regulation of cell migration by PTEN. Biochem. Soc. Trans.

[5] Backman S., Stambolic V., Mak T. (2002). PTEN function in mammalian cell size regulation. Curr. Opin. Neurobiol.

[6] Ji S.P., Zhang Y., Van Cleemput J., Jiang W., Liao M., Li L., Wan Q., Backstrom J.R., Zhang X. (2006). Disruption of PTEN coupling with 5-HT2C receptors suppresses behavioral responses induced by drugs of abuse. Nat. Med.

[7] Kwon C.H., Zhu X., Zhang J., Knoop L.L., Tharp R., Smeyne R.J., Eberhart C.G., Burger P.C., Baker S.J. (2001). Pten regulates neuronal soma size: A mouse model of Lhermitte-Duclos disease. Nat. Genet.

[8] Omori N., Jin G., Li F., Zhang W.R., Wang S.J., Hamakawa Y., Nagano I., Manabe Y., Shoji M., Abe K. (2002). Enhanced phosphorylation of PTEN in rat brain after transient middle cerebral artery occlusion. Brain Res.

[9] Walker C.L., Walker M.J., Liu N.K., Risberg E.C., Gao X., Chen J., Xu X.M. (2012). Systemic bisperoxovanadium activates Akt/mTOR, reduces autophagy, and enhances recovery following cervical spinal cord injury. PLoS One.

[10] Huang X., Zhang H., Yang J., Wu J., McMahon J., Lin Y., Cao Z., Gruenthal M., Huang Y. (2010). Pharmacological inhibition of the mammalian target of rapamycin pathway suppresses acquired epilepsy. Neurobiol. Dis.

[11] Choi Y.C., Lee J.H., Hong K.W., Lee K.S. (2004). 17 Beta-estradiol prevents focal cerebral ischemic damages via activation of Akt and CREB in association with reduced PTEN phosphorylation in rats. Fundam. Clin. Pharmacol.

[12] Schmid A.C., Byrne R.D., Vilar R., Woscholski R. (2004). Bisperoxovanadium compounds are potent PTEN inhibitors. FEBS Lett.

[13] Shackelford D.A., Yeh R.Y. (2006). Modulation of ERK and JNK activity by transient forebrain ischemia in rats. J. Neurosci. Res.

[14] Wahl F., Allix M., Plotkine M., Boulu R.G. (1992). Neurological and behavioral outcomes of focal cerebral ischemia in rats. Stroke.

[15] Yang Y., Li Q., Miyashita H., Howlett W., Siddiqui M., Shuaib A. (2000). Usefulness of postischemic thrombolysis with or without neuroprotection in a focal embolic model of cerebral ischemia. J. Neurosurg.

[16] Brunet A., Bonni A., Zigmond M.J., Lin M.Z., Juo P., Hu L.S., Anderson M.J., Arden K.C., Blenis J., Greenberg M.E. (1999). Akt promotes cell survival by phosphorylating and inhibiting a Forkhead transcription factor. Cell.

[17] Datta S.R., Dudek H., Tao X., Masters S., Fu H., Gotoh Y., Greenberg M.E. (1997). Akt phosphorylation of BAD couples survival signals to the cell-intrinsic death machinery. Cell.

[18] Hetman M., Cavanaugh J.E., Kimelman D., Xia Z. (2000). Role of glycogen synthase kinase-3beta in neuronal apoptosis induced by trophic withdrawal. J. Neurosci.

[19] Cantley L.C., Neel B.G. (1999). New insights into tumor suppression: PTEN suppresses tumor formation by restraining the phosphoinositide 3-kinase/AKT pathway. Proc. Natl. Acad. Sci. USA.

[20] Liu K., Lu Y., Lee J.K., Samara R., Willenberg R., Sears-Kraxberger I., Tedeschi A., Park K.K., Jin D., Cai B. (2010). PTEN deletion enhances the regenerative ability of adult corticospinal neurons. Nat. Neurosci.

[21] Gary D.S., Mattson M.P. (2002). PTEN regulates Akt kinase activity in hippocampal neurons and increases their sensitivity to glutamate and apoptosis. Neuromol. Med.

[22] Ruan H., Li J., Ren S., Gao J., Li G., Kim R., Wu H., Wang Y. (2009). Inducible and cardiac specific PTEN inactivation protects ischemia/reperfusion injury. J. Mol. Cell. Cardiol.

[23] Liao J., Marumoto T., Yamaguchi S., Okano S., Takeda N., Sakamoto C., Kawano H., Nii T., Miyamato S., Nagai Y. (2013). Inhibition of PTEN Tumor Suppressor Promotes the Generation of Induced Pluripotent Stem Cells. Mol. Ther..

[24] Sun F., Park K.K., Belin S., Wang D., Lu T., Chen G., Zhang K., Yeung C., Feng G., Yankner B.A., He Z. (2011). Sustained axon regeneration induced by co-deletion of PTEN and SOCS3. Nature.

[25] Longa E.Z., Weinstein P.R., Carlson S., Cummins R. (1989). Reversible middle cerebral artery occlusion without craniectomy in rats. Stroke.

[26] Du W., Huang J., Yao H., Zhou K., Duan B., Wang Y. (2010). Inhibition of TRPC6 degradation suppresses ischemic brain damage in rats. J. Clin. Invest.

[27] Swanson R.A., Morton M.T., Tsao-Wu G., Savalos R.A., Davidson C., Sharp F.R. (1990). A semiautomated method for measuring brain infarct volume. J. Cereb. Blood Flow Metab..

[28] Saito A., Hayashi T., Okuno S., Ferrand-Drake M., Chan P.H. (2003). Overexpression of copper/zinc superoxide dismutase in transgenic mice protects against neuronal cell death after transient focal ischemia by blocking activation of the Bad cell death signaling pathway. J. Neurosci.

[29] Ashwal S., Tone B., Tian H.R., Cole D.J., Pearce W.J. (1998). Core and penumbral nitric oxide synthase activity during cerebral ischemia and reperfusion. Stroke.

